# Five-State Extended Kalman Filter for Estimation of Speed over Ground (SOG), Course over Ground (COG) and Course Rate of Unmanned Surface Vehicles (USVs): Experimental Results

**DOI:** 10.3390/s21237910

**Published:** 2021-11-27

**Authors:** Sindre Fossen, Thor I. Fossen

**Affiliations:** 1 Maritime Robotics AS, Brattørkaia 11, Pirterminalen, 7010 Trondheim, Norway; sindre.fossen@maritimerobotics.com; 2 Department of Engineering Cybernetics, Norwegian University of Science and Technology (NTNU), 7491 Trondheim, Norway

**Keywords:** unmanned surface vehicle (USV), Kalman filter, course autopilot, course over ground, speed over ground

## Abstract

Small USVs are usually equipped with a low-cost navigation sensor suite consisting of a global navigation satellite system (GNSS) receiver and a magnetic compass. Unfortunately, the magnetic compass is highly susceptible to electromagnetic disturbances. Hence, it should not be used in safety-critical autopilot systems. A gyrocompass, however, is highly reliable, but it is too expensive for most USV systems. It is tempting to compute the heading angle by using two GNSS antennas on the same receiver. Unfortunately, for small USV systems, the distance between the antennas is very small, requiring that an RTK GNSS receiver is used. The drawback of the RTK solution is that it suffers from dropouts due to ionospheric disturbances, multipath, interference, etc. For safety-critical applications, a more robust approach is to estimate the course angle to avoid using the heading angle during path following. The main result of this article is a five-state extended Kalman filter (EKF) aided by GNSS latitude-longitude measurements for estimation of the course over ground (COG), speed over ground (SOG), and course rate. These are the primary signals needed to implement a course autopilot system onboard a USV. The proposed algorithm is computationally efficient and easy to implement since only four EKF covariance parameters must be specified. The parameters need to be calibrated for different GNSS receivers and vehicle types, but they are not sensitive to the working conditions. Another advantage of the EKF is that the autopilot does not need to use the COG and SOG measurements from the GNSS receiver, which have varying quality and reliability. It is also straightforward to add complementary sensors such as a Doppler Velocity Log (DVL) to the EKF to improve the performance further. Finally, the performance of the five-state EKF is demonstrated by experimental testing of two commercial USV systems.

## 1. Introduction

**Figure 1:**
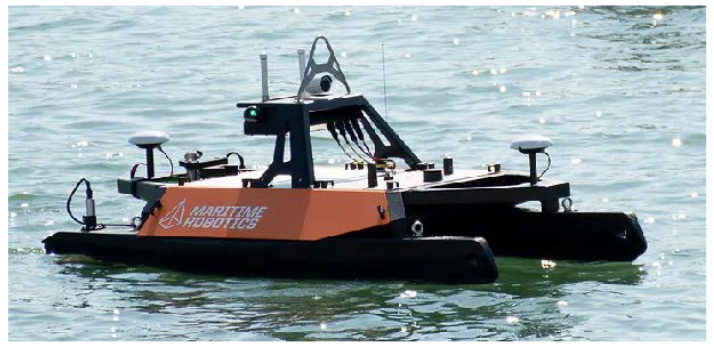
The Maritime Robotics Otter Pro USV (dimensions 2.00 × 1.07 × 0.82 m and weight 65 kg) [3].

**Figure 2:**
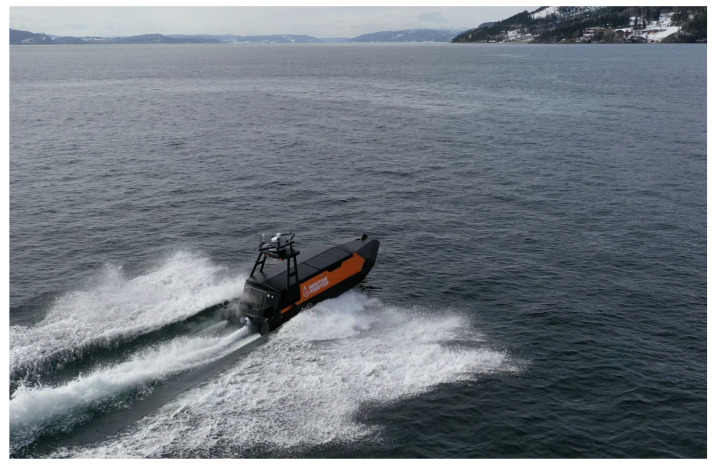
The Maritime Robotics Mariner USV (dimensions 5.95 × 2.05 × 2.00 m and weight 1900 kg) [3].

Commercial unmanned surface vehicle (USV) systems are used in many operations such as harbor inspection, surveillance, mapping, data acquisition, oceanography, etc. (see Figure 1 and Figure 2). This creates a need for low-cost sensor systems to operate a USV safely with satisfactory performance. The autopilot system is a critical component that is used for turning and path following. Both heading and course autopilots can be used for this purpose. Course autopilots are the preferred solution during path following. However, for stationkeeping it is necessary to control the heading angle. The reason for this is that the course angle is not defined at zero speed.

Ships are usually equipped with a gyrocompass, which is a nonmagnetic compass able to find true North using a fast-spinning gyroscope (Fossen [1]). The gyrocompass gives a highly accurate measurement of the heading (yaw) angle, but it is too expensive to be used in small USV systems. Hence, it is tempting to use a low-cost magnetic compass for navigation and maneuvering. This is not straightforward since a magnetic compass is susceptible to magnetic disturbances produced by electromagnetic devices such as propellers and thrusters. In addition, the magnetic field of the Earth is not perfectly aligned but skewed along the Earth’s rotational axis. The skew or bias is called declination, and it must be compensated for when navigating.

An alternative measurement to the compass could be to use two GNSS antennas on the same receiver with a known offset vector to compute the heading angle. The accuracy is further improved by using real-time kinematic (RTK) GNSS positioning (Farrell [2]). This is the preferred solution for small USVs since the offset vector can be small. It is well known that the RTK GNSS receivers are sensitive to ionospheric disturbances, multipath, loss of signals, the number of available satellites, etc., so the reliability can, in many cases, be unsatisfactory.

The scope of this article is to derive a robust state estimator for SOG, COG, and course rate such that these signals can be used to design a USV course autopilot for path following. The state estimator should only use the North–East positions, alternatively the latitude and longitude, of a GNSS receiver. If the course angle is available as a direct measurement or an estimate, it is straightforward to design a proportional-integral-derivative (PID) controller for course control; see Fossen [1].

The dynamics of a USV moving along a path or a trajectory can be modeled by using 2D target-tracking models (Li and Jilkov [4]). The simplest models for a target-tracking maneuver are the white-noise constant velocity (CV) and constant acceleration (CA) models (Bar-Shalom et al. [5]). These models are based on the assumption that the target speed or acceleration are independent processes driven by Gaussian white noise. The Otter and Mariner USVs shown in Figure 1 and Figure 2, respectively, are not able to produce aggressive linear accelerations. Hence, the CV model is well suited to describe the vehicle’s speed *U* during path-following control, since *U* is nearly constant even though the USV experiences small linear accelerations. Analysis of automatic identification system (AIS) data confirms this (Siegert et al. [6]). However, both USVs have propellers that can turn the vehicles quite fast. This suggests that the course angle χ should be modeled by a CA model. Consequently, the target-tracking models are chosen as a combination of the CV and CA models according to

(1) $$\begin{array}{cc} \dot{U} & =w_{1} \end{array}$$

(2) $$\begin{array}{cc} \dot{\chi } & =\omega_{\chi } \end{array}$$

(3) $$\begin{array}{cc} \dot{\omega_{\chi }} & =w_{2} \end{array}$$

where $w_{1}$ and $w_{2}$ are Gaussian white-noise processes. The models (1)–(3) will be used as a basis for the EKF presented in Section 3.

The main result of this article is a five-state EKF (Appendix A) aided by GNSS positions or latitude–longitude measurements, which is intended for course and path-following control. The EKF can also be used to process AIS measurements (Fossen and Fossen [7]) with the purpose of ship motion prediction. The algorithm is computationally efficient and easy to implement. The main advantage of the EKF is that vendors do not need to use COG and SOG measurements from GNSS receivers, which have varying quality. In addition, the algorithm produces an accurate estimate of the course rate, which is an important signal when implementing PID course autopilot systems.

The remainder of this article is organized as follows: Section 2 presents the kinematic equations of a surface vehicle, while Section 3 contains the EKF for COG, SOG, and course rate estimation. Section 4 describes a method for USV course autopilot design. Section 5 includes a simulation study of a small USV under course autopilot control, while Section 6 and Section 7 present the experimental results using two commercial USV systems. The discussion and concluding remarks are drawn in Section 8 and Section 9.

## 2. Kinematics

**Figure 3:**
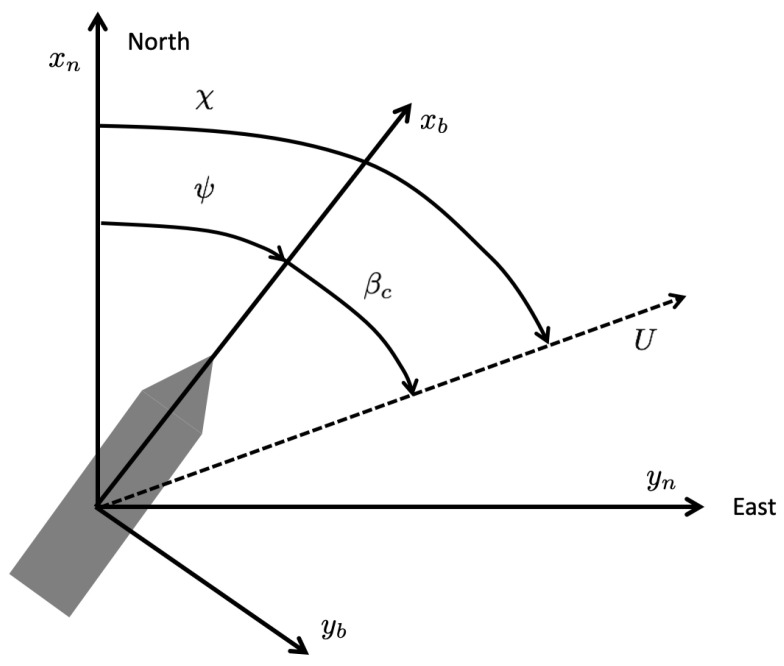
Heading, course, and crab angles.

The relationship between the angular variables *course, heading*, and *crab angle* is important for maneuvering of a USV in the horizontal plane. The terms course and heading are used interchangeably in much of the literature on marine systems, and this leads to confusion. Let the BODY and North-East-Down (NED) reference frames in Figure 3 be denoted by $\left\{b\right\}=\left(x_{b},\;y_{b},\;z_{b}\right)$ and $\left\{n\right\}=\left(x_{n},\;y_{n},\;z_{n}\right)$, respectively.

**Definition** **1.** ***(Yaw or heading angle ψ)** The angle ψ from the $x_{n}$ axis (true North) to the $x_{b}$ axis of the USV, positive rotation about the $z_{n}$ axis by the right-hand screw convention.*

The heading angle is usually measured by using a magnetic compass, gyrocompass or two GNSS receivers; see Gade [8] for a discussion of methods. The heading angle is well defined for zero speed such that it is possible to design a *heading autopilot* to maintain constant heading during stationkeeping and transit. However, during transit it is common to use a *course autopilot* for path following.

**Definition** **2.** ***(Course angle χ)** The angle χ from the $x_{n}$ axis (true North) to the velocity vector of the USV, positive rotation about the $z_{n}$ axis by the right-hand screw convention.*

Note that the course angle is only defined for positive speed. The North–East positions $\left(x^{n},\;y^{n}\right)$ of a USV can be described by (Fossen [1]),

(4) $$\begin{array}{cc} {\dot{x}}^{n} & =u\cos(\psi )-v\sin(\psi ) \end{array}$$

(5) $$\begin{array}{cc} {\dot{y}}^{n} & =u\sin(\psi )+v\cos(\psi ) \end{array}$$

where (u,v) are the surge and sway velocities, respectively. These equations can be expressed in *amplitude-phase* form by

(6) $$\begin{array}{cc} {\dot{x}}^{n} & =U\cos\left(\psi +\beta_{c}\right):=U\cos\left(\chi \right) \end{array}$$

(7) $$\begin{array}{cc} {\dot{y}}^{n} & =U\sin\left(\psi +\beta_{c}\right):=U\sin\left(\chi \right) \end{array}$$

where the course angle is defined as

(8) $$\chi :=\psi +\beta_{c}$$

Furthermore, the amplitude *U* and phase variable $\beta_{c}$ are

(9) $$\begin{array}{cc} U & =\sqrt{u^{2}+v^{2}} \end{array}$$

(10) $$\begin{array}{cc} \beta_{c} & =\mathrm{atan}\left(\frac{v}{u}\right)=\sin^{-1}\left(\frac{v}{U}\right) \end{array}$$

Note that *U* is the speed in the horizontal plane, and $\beta_{c}$ is the crab angle.

**Definition** **3.** ***(Crab angle $\beta_{c}$)** The angle $\beta_{c}$ from the $x_{b}$ axis to the velocity vector of the USV, positive rotation about the $z_{b}$ axis by the right-hand screw convention.*

## 3. EKF for SOG, COG and Course Rate

The primary objective of the EKF is to compute accurate estimates of the SOG, COG, and course rate of the USV when moving along a path. The dynamic model of the EKF ensures that old GNSS position measurements are used to compute the estimates. Since the path is not parametrized and the heading angle is unknown, the only information during path following is the measured GNSS position, alternatively the latitude-longitude pair.

### 3.1. Five-State EKF: North-East Position Measurements

The North–East positions $\left(x^{n},y^{n}\right)$ of the USV are given by (6) and (7), while the speed and the course angle are modeled by the CV and CA models (1)–(3). The resulting state–space model expressed in North–East coordinates is

(11) $$\begin{array}{cc} {\dot{x}}^{n} & =U\cos(\chi ) \end{array}$$

(12) $$\begin{array}{cc} {\dot{y}}^{n} & =U\sin(\chi ) \end{array}$$

(13) $$\begin{array}{cc} \dot{U} & =-\alpha_{1}U+w_{1} \end{array}$$

Subjected to(2)

(14) $$\begin{array}{cc} \dot{\omega_{\chi }} & =-\alpha_{2}\omega_{\chi }+w_{2} \end{array}$$

where $w_{1}$ and $w_{2}$ are Gaussian white-noise process noise. Two small constants $\alpha_{1}\geq 0$ and $\alpha_{2}\geq 0$ have been added to the model to ensure that *U* and $\omega_{\chi }$ converge to zero during stationkeeping. Equations (13) and (14) are referred to as *Singer models* [9] in the target-tracking community (Li and Jilkov [4]).

The GNSS measurement equations associated with (11)–(14) are

(15) $$\begin{array}{cc} y_{1} & =x^{n}+\varepsilon_{1} \end{array}$$

(16) $$\begin{array}{cc} y_{2} & =y^{n}+\varepsilon_{2} \end{array}$$

where $\varepsilon_{1}$ and $\varepsilon_{2}$ are Gaussian white-noise measurement noise. For the speed Equation (13), simulation studies revealed that the CV model with the Singer modification $\alpha_{1}=0.01$ was most accurate for USVs since the speed is *nearly-constant* most of the time, i.e., small linear accelerations. Turning was accurately described by using the CA model with $\alpha_{2}=0.1$. This gave satisfactory course rate estimates.

The discrete-time representation of (11)–(14) is obtained by Euler’s method

(17) $$\begin{array}{cc} x^{n}\left[k+1\right] & =x^{n}\left[k\right]+h\;U\left[k\right]\cos\left(\chi \left[k\right]\right) \end{array}$$

(18) $$\begin{array}{cc} y^{n}\left[k+1\right] & =y^{n}\left[k\right]+h\;U\left[k\right]\sin\left(\chi \left[k\right]\right) \end{array}$$

(19) $$\begin{array}{cc} U[k+1] & =\left(1-h\alpha_{1}\right)U\left[k\right]+h\;w_{1}\left[k\right] \end{array}$$

(20) $$\begin{array}{cc} \chi [k+1] & =\chi \left[k\right]+h\;\omega_{\chi }\left[k\right] \end{array}$$

(21) $$\begin{array}{cc} \omega_{\chi }\left[k+1\right] & =\left(1-h\alpha_{2}\right)\omega_{\chi }\left[k\right]+h\;w_{2}\left[k\right] \end{array}$$

where *h* is used to denote the sampling time. The discrete-time measurement equations are

(22) $$\begin{array}{cc} y_{1}\left[k\right] & =x^{n}\left[k\right]+\varepsilon_{1}\left[k\right] \end{array}$$

(23) $$\begin{array}{cc} y_{2}\left[k\right] & =y^{n}\left[k\right]+\varepsilon_{2}\left[k\right] \end{array}$$

Consequently, the discrete-time state–space model becomes

(24) $$\begin{array}{cc} x[k+1] & =A_{d}\;x\left[k\right]+E_{d}w\left[k\right] \end{array}$$

(25) $$\begin{array}{cc} y[k] & =C_{d}\;x\left[k\right]+\varepsilon \left[k\right] \end{array}$$

where

(26) $$\begin{array}{cc} x & ={\left[x^{n},y^{n},U,\chi ,\omega_{\chi }\right]}^{⊤} \end{array}$$

(27) $$\begin{array}{cc} y & ={\left[x^{n},y^{n}\right]}^{⊤} \end{array}$$

(28) $$\begin{array}{cc} w & ={\left[w_{1},w_{2}\right]}^{⊤} \end{array}$$

(29) $$\begin{array}{cc} \varepsilon & ={\left[\varepsilon_{1},\varepsilon_{2}\right]}^{⊤} \end{array}$$

The Jacobians are

(30) $$A_{d}=\left[\begin{array}{ccccc} 1 & 0 & h\cos({\hat{x}}_{4}\left[k\right]) & -h\;{\hat{x}}_{3}\left[k\right]\sin\left({\hat{x}}_{4}\left[k\right]\right) & 0\\ 0 & 1 & h\sin({\hat{x}}_{4}\left[k\right]) & h\;{\hat{x}}_{3}\left[k\right]\cos\left({\hat{x}}_{4}\left[k\right]\right) & 0\\ 0 & 0 & 1-h\alpha_{1} & 0 & 0\\ 0 & 0 & 0 & 1 & h\\ 0 & 0 & 0 & 0 & 1-h\alpha_{2} \end{array}\right]$$

(31) $$E_{d}=\left[\begin{array}{cc} 0 & 0\\ 0 & 0\\ h & 0\\ 0 & 0\\ 0 & h \end{array}\right],\;C_{d}=\left[\begin{array}{ccccc} 1 & 0 & 0 & 0 & 0\\ 0 & 1 & 0 & 0 & 0 \end{array}\right]$$

The resulting EKF algorithm (see Brown and Hwang [10]) is summarized in Table 1 where $h\left({\hat{x}}^{-}\left[k\right]\right)=C_{d}\left[k\right]{\hat{x}}^{-}\left[k\right]$, and $Q_{d}\left[k\right]$ and $R_{d}\left[k\right]$ are the covariance matrices for the process and measurement noises. The *a priori* state and covariance matrix estimates (before update) are denoted $\left({\hat{x}}^{-}\left[k\right],\;{\hat{P}}^{-}\left[k\right]\right)$, while the *a posteriori* state and covariance matrix estimates (after update) are denoted by $\left(\hat{x}\left[k\right],\;\hat{P}\left[k\right]\right)$.

### 3.2. Five-State EKF: Latitude and Longitude Measurements

GNSS receivers output latitude, μ, longitude, *l*, and elevation, *h*, using the World Geodetic System (WGS-84) ellipsoid as reference system [11]. For vehicles operating on the sea surface, we chose the height above the reference geoid as h=0. The  coordinate origin is conveniently fixed at a point on Earth’s surface, specified by its latitude and longitude pair $\left(\mu_{0},l_{0}\right)$. The Earth radius of curvature in the prime vertical, $R_{N}$, and the radius of curvature in the meridian, $R_{M}$, are (Farrell [2]),

(32) $$\begin{array}{cc} R_{N} & =\frac{r_{e}}{\sqrt{1-e^{2}\sin^{2}\left(\mu_{0}\right)}} \end{array}$$

(33) $$\begin{array}{cc} R_{M} & =R_{N}\frac{1-e^{2}}{\sqrt{1-e^{2}\sin^{2}\left(\mu_{0}\right)}} \end{array}$$

where $r_{e}=6,378,137\;m$ is the semi-minor axis (equatorial radius), and e=0.0818 is the Earth eccentricity (WGS-84). Consequently, the latitude and longitude dynamics are

(34) $$\begin{array}{cc} \dot{\mu } & =\frac{1}{R_{M}}v_{N}=\frac{1}{R_{M}}U\cos\left(\chi \right) \end{array}$$

(35) $$\begin{array}{cc} \dot{l} & =\frac{1}{R_{N}\cos\left(\mu \right)}v_{E}=\frac{1}{R_{N}\cos\left(\mu \right)}U\sin\left(\chi \right) \end{array}$$

where $v_{N}$ and $v_{E}$ are the North–East velocities of the vehicle. The discrete-time EKF model for longitude and latitude are obtained by Euler’s method

(36) $$\begin{array}{cc} \mu [k+1] & =\mu \left[k\right]+h\;\frac{1}{R_{M}}U\left[k\right]\cos\left(\chi \left[k\right]\right) \end{array}$$

(37) $$\begin{array}{cc} l[k+1] & =l\left[k\right]+h\;\frac{1}{R_{N}\cos\left(\mu \left[k\right]\right)}U\left[k\right]\sin\left(\chi \left[k\right]\right) \end{array}$$

Subjected to(19)

Subjected to(20)

Subjected to(21)

The state–space representation is

Subjected to(24)

Subjected to(25)

where

(38) $$\begin{array}{cc} x & ={\left[\mu ,l,U,\chi ,\omega_{\chi }\right]}^{⊤} \end{array}$$

(39) $$\begin{array}{cc} y & ={\left[\mu ,l\right]}^{⊤} \end{array}$$

(40) $$\begin{array}{cc} w & ={\left[w_{1},w_{2}\right]}^{⊤} \end{array}$$

(41) $$\begin{array}{cc} \varepsilon & ={\left[\varepsilon_{1},\varepsilon_{2}\right]}^{⊤} \end{array}$$

The Jacobians are

(42) $$A_{d}=\left[\begin{array}{ccccc} 1 & 0 & h\frac{\cos({\hat{x}}_{4}\left[k\right])}{R_{M}} & -h\frac{{\hat{x}}_{3}\left[k\right]\sin\left({\hat{x}}_{4}\left[k\right]\right)}{R_{M}} & 0\\ A_{21} & 1 & h\frac{\sin({\hat{x}}_{4}\left[k\right])}{R_{N}\cos\left({\hat{x}}_{1}\left[k\right]\right)} & h\frac{{\hat{x}}_{3}\left[k\right]\cos\left({\hat{x}}_{4}\left[k\right]\right)}{R_{N}\cos\left({\hat{x}}_{1}\left[k\right]\right)} & 0\\ 0 & 0 & 1-h\alpha_{1} & 0 & 0\\ 0 & 0 & 0 & 1 & h\\ 0 & 0 & 0 & 0 & 1-h\alpha_{2} \end{array}\right]$$

where

(43) $$A_{21}=h\;\frac{{\hat{x}}_{3}\left[k\right]\sin\left({\hat{x}}_{4}\left[k\right]\right)\tan\left({\hat{x}}_{1}\left[k\right]\right)}{R_{N}\cos\left({\hat{x}}_{1}\left[k\right]\right)}$$

and

Subjected to(31)

## 4. USV Course Autopilot Design

The course angle dynamics can be approximated by a first-order model (Nomoto [12])

Subjected to(2)

(44) $$\begin{array}{cc} {\dot{\omega }}_{\chi }+\frac{1}{T}\omega_{\chi }\; & =\frac{K}{T}\tau_{N}+d_{\omega } \end{array}$$

where *T* is the time constant in yaw, and *K* is implicitly defined by the ratio

(45) $$\frac{K}{T}=\frac{1}{I_{z}-N_{\dot{r}}}$$

Here, $I_{z}$ is the moment of inertia, $-N_{\dot{r}}$ is the hydrodynamic added moment of inertia, $\tau_{N}$ is the yaw moment, and $d_{\omega }$ is a time-varying disturbance due to unmodeled dynamics and environmental disturbances. In practice, $d_{\omega }$ will be a nearly constant drift term, which can be compensated by an integral controller.

The Nomoto gain and time constants *K* and *T*, respectively, can be determined by a maneuvering test. e.g., a turning circle or a zigzag test (Fossen [1]). The course autopilot was chosen as a PID controller with reference feedforward

(46) $$\tau_{N}=\tau_{FF}-K_{p}\left(\mathrm{ssa}\left(\tilde{\chi }\right)+\frac{1}{T_{i}}\int_{0}^{t}\mathrm{ssa}\left(\tilde{\chi }\right)d\tau +T_{d}{\tilde{\omega }}_{\chi }\right)$$

where $\tau_{N}$ is the commanded yaw moment, $K_{p}$ is the proportional gain, $T_{d}$ is the derivative time constant, and $T_{i}$ is the integral time constant. The course angular velocity tracking error is denoted by ${\tilde{\omega }}_{\chi }=\omega_{\chi }-\omega_{\chi_{d}}$, where the subscript *d* denotes the desired value. The unconstrained course angle tracking error $\tilde{\chi }=\chi -\chi_{d}$ is mapped to the interval [−π,π) using the operator ssa:R→[−π,π) representing the smallest-signed angle (SSA) or difference between the two angles χ and $\chi_{d}$. The Marine Systems Simulator (MSS) Matlab implementation is (Fossen and Perez [13]),

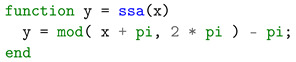



The reference feedforward signal was chosen as

(47) $$\tau_{FF}=\frac{T}{K}{\dot{\omega }}_{\chi_{d}}+\frac{1}{K}\omega_{\chi_{d}}$$

where $\omega_{\chi_{d}}$ and ${\dot{\omega }}_{\chi_{d}}$ are the desired angular velocity and acceleration, respectively. The resulting closed-loop system is

(48) $$\ddot{\tilde{\chi }}+\left(\frac{1}{T}+\frac{KK_{p}T_{d}}{T}\right)\dot{\tilde{\chi }}+\frac{KK_{p}}{T}\mathrm{ssa}\left(\tilde{\chi }\right)+\frac{KK_{p}}{TT_{i}}\int_{0}^{t}\;\;\;\mathrm{ssa}\left(\tilde{\chi }\right)d\tau =d_{\omega }$$

The PID controller gains can be determined by pole placement with $\omega_{n}$ and ζ as design parameters. This gives

(49) $$\begin{array}{cc} \frac{KK_{p}}{T}:=\omega_{n}^{2} & \Rightarrow K_{p}=\frac{T}{K}\omega_{n}^{2} \end{array}$$

(50) $$\begin{array}{cc} \frac{1}{T}+\frac{KK_{p}T_{d}}{T}:=2\zeta \omega_{n} & \Rightarrow T_{d}=\frac{T}{KK_{p}}\left(2\zeta \omega_{n}-\frac{1}{T}\right) \end{array}$$

The integrator time constant was chosen such that $1/T_{i}=\omega_{n}/10$. In other words,

(51) $$T_{i}=\frac{10}{\omega_{n}}$$

This guarantees that the tracking errors $\tilde{\chi }$ and ${\tilde{\omega }}_{\chi }$ converge exponentially to zero under the assumption that ${\dot{d}}_{\omega }=0$. Global exponential stability cannot be proven since the course angle error is defined on [−π,π) and not R as shown by Bhat and Bernstein [14].

## 5. Simulation Study of the Otter USV

In the simulation study, a mathematical model of the Maritime Robotics Otter USV, length L=2.0m, was used. The model is included in the Matlab MSS toolbox (Fossen and Perez [13]) as a function

xdot = otter(x,n,mp,rp,V_c,beta_c)

which returns the time derivative, xdot, of the state vector

x = [u,v,w,p,q,r,x,y,z,phi,theta,psi]’

The inputs are the left and right propeller shaft speeds n = [n1,n2]’, the mass of the payload, m_p, the location of the payload, r_p = [x_p,y_p,z_p]’, ocean current speed, V_c, and ocean current direction, beta_c. The toolbox also has a Simulink block for numerical integration of the m-file function.

### Estimation of SOG, COG and Course Rate during Course Autopilot Control

In the simulation study, the five-state EKF in Section 3.1 was used to estimate the course angle and course rate, which are the feedback signals needed to implement the course autopilot (46). The filter sampling frequency was chosen as 50Hz, while the GNSS position measurements were received at 5Hz.

The MSS Otter USV model is controlled by two propellers with shaft speeds $n_{1}$ and $n_{2}$ in rad/s. The propellers produce a surge force $\tau_{X}$ and a yaw moment $\tau_{N}$ according to

(52) $$\begin{array}{cc} \left[\begin{array}{c} \tau_{X}\\ \tau_{N} \end{array}\right] & =k\left[\begin{array}{cc} 1 & 1\\ -l_{1} & -l_{2} \end{array}\right]\left[\begin{array}{c} |n_{1}|n_{1}\\ |n_{2}|n_{2} \end{array}\right] \end{array}$$

where $l_{1}=-0.395\;m$, $l_{2}=0.395\;m$ and k=0.0111kgm is the propeller gain.

The operator specifies the desired force $\tau_{X}$ in the surge direction, while the course autopilot (46) computes the desired yaw moment $\tau_{N}$. The control allocation problem is solved by applying the inverse mapping

(53) $$\begin{array}{cc} \left[\begin{array}{c} u_{1}\\ u_{2} \end{array}\right] & =\frac{1}{k}{\left[\begin{array}{cc} 1 & 1\\ -l_{1} & -l_{2} \end{array}\right]}^{-1}\left[\begin{array}{c} \tau_{X}\\ \tau_{N} \end{array}\right] \end{array}$$

(54) $$\begin{array}{cc} n_{1} & =\mathrm{sgn}\left(u_{1}\right)\sqrt{|u_{1}|} \end{array}$$

(55) $$\begin{array}{cc} n_{2} & =\mathrm{sgn}\left(u_{2}\right)\sqrt{|u_{2}|} \end{array}$$

The Nomoto time and gain constants of the Otter USV were estimated to

(56) $$\begin{array}{cc} T & =1.0\;s \end{array}$$

(57) $$\begin{array}{cc} K & =0.0242\;\mathrm{kg}m^{2}s \end{array}$$

The course autopilot system was implemented as

(58) $$\begin{array}{cc} \tau_{X} & =\mathrm{Pilot}\mathrm{input} \end{array}$$

Subjected to(46)

where the controller gains are determined by (49)–(51) by specifying ζ=1.0 and $\omega_{n}=1.5\;\mathrm{rad}/s$. This yields

(59) $$K_{p}=93.15,\;T_{d}=0.89,\;T_{i}=6.67$$

**Figure 4:**
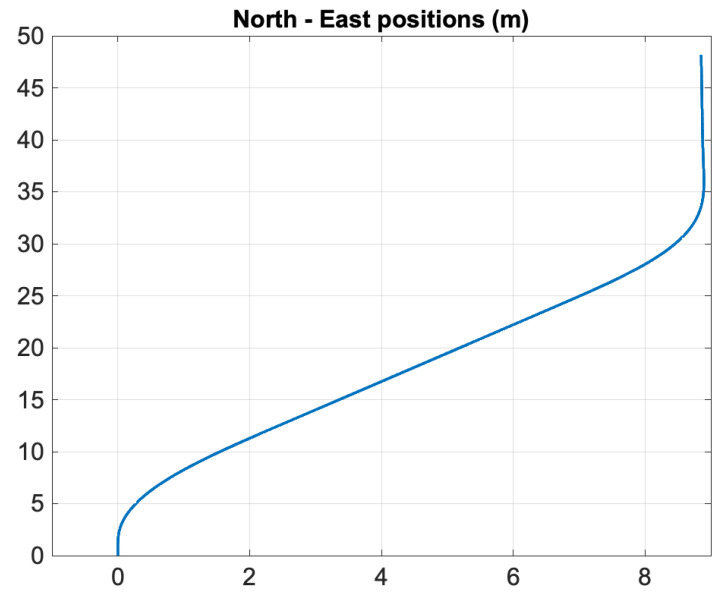
Simulation study: North–East positions of the USV for a 20 degrees course command, which is changed back to 0 degrees at time t=20 s.

**Figure 5:**
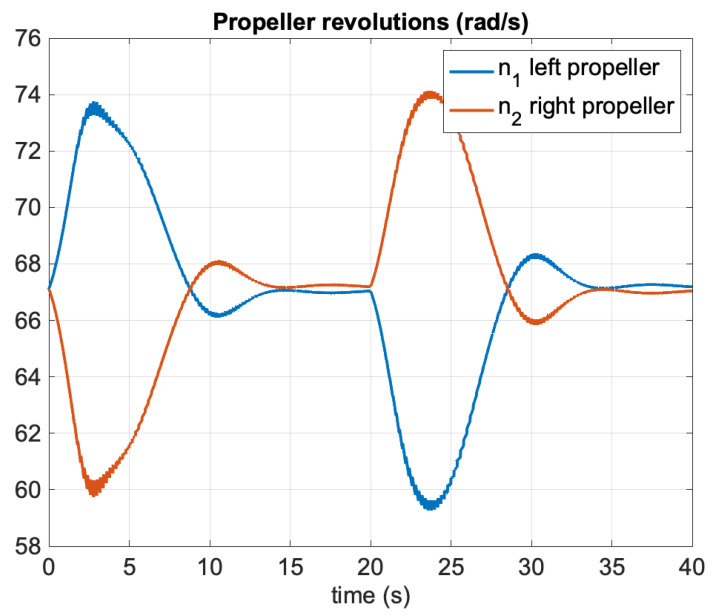
Simulation study: Propeller revolutions $n_{1}$ and $n_{2}$ versus time during autopilot control.

**Figure 6:**
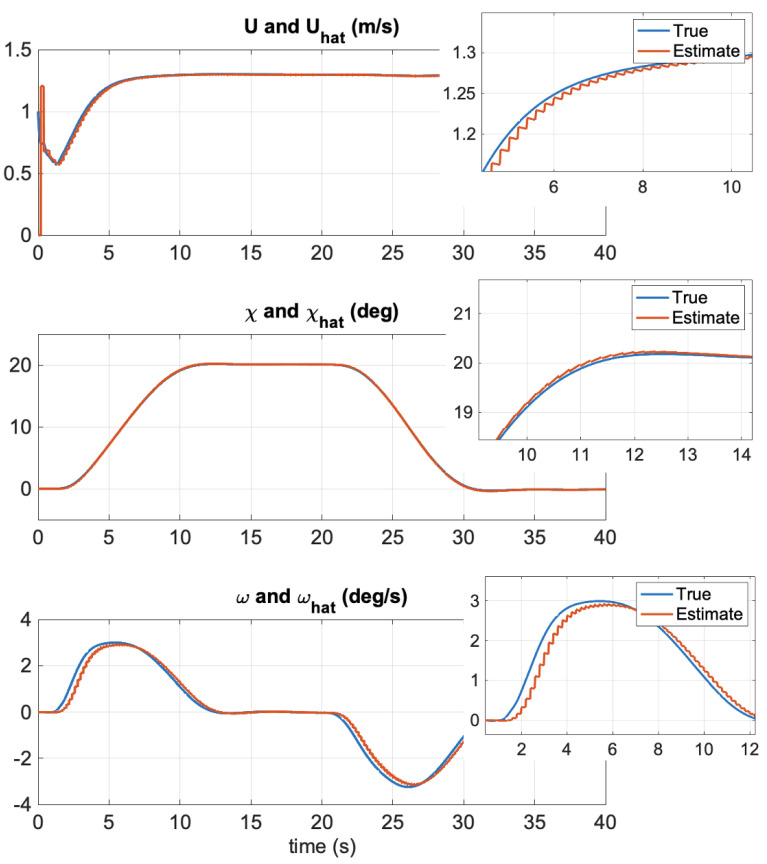
Simulation study: Estimated SOG, COG, and course rate versus time. The zoomed plots to the right show the slow GNSS rate (5 Hz) compared to the EKF sampling time (50 Hz). The autopilot performed a 20 degrees course command, which was returned to 0 degrees at time t=20 s.

Figure 4 shows the North–East positions during autopilot control. The propeller commands are shown in Figure 5 where the control allocation algorithm (53)–(55) was applied. Figure 6 clearly demonstrates that the EKF was able to estimate the unmeasured states *U*, χ, and $\omega_{\chi }$ quite accurately. The zoomed windows show the 5 Hz slow update rate of the filter (GNSS measurements frequency) compared to the 50 Hz sampling frequency of the predictor.

## 6. Experiments with the Mariner USV

**Figure 7:**
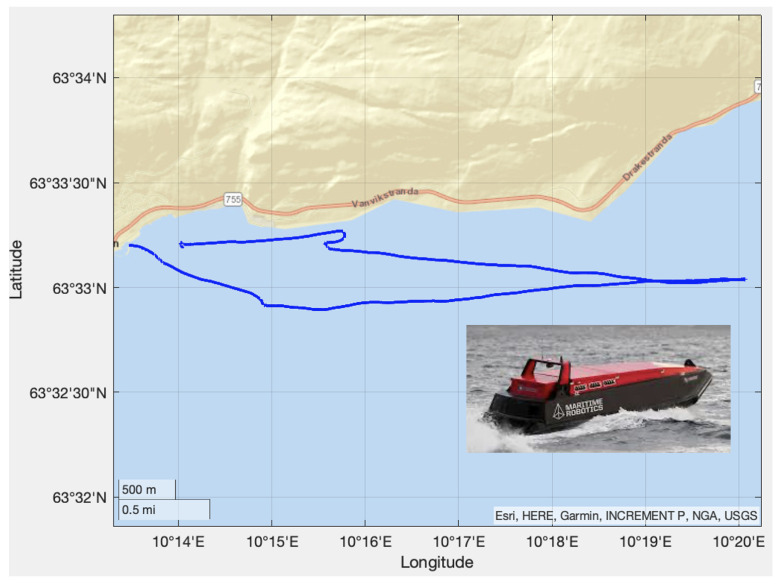
The blue line shows the path of the Mariner USV. The starting point is Vanvikan located north of Trondheim in the Trondheim fjord at $63^{\circ }\;33^{\prime }\;11.23^{\prime }\;N$ and $10^{\circ }\;14^{\prime }\;2.90^{\prime \prime }\;E$.

In the first experiment, the Mariner USV was used. The geographical location is shown in Figure 7. The blue line indicates the traveled path in the Trondheim fjord, Norway. The experiments were performed in sea state 2 corresponding to wave amplitudes below 0.5 m. Latitude and longitude were measured using a u-blox NEO-M8Q GNSS receiver at 5 Hz [15], while the SOG and COG measurements were complementary measurements used to benchmark the EKF. The accuracy of the NEO-MQ8 in the horizontal plane was 2.5 m when using GPS/Glonass.

**Figure 8:**
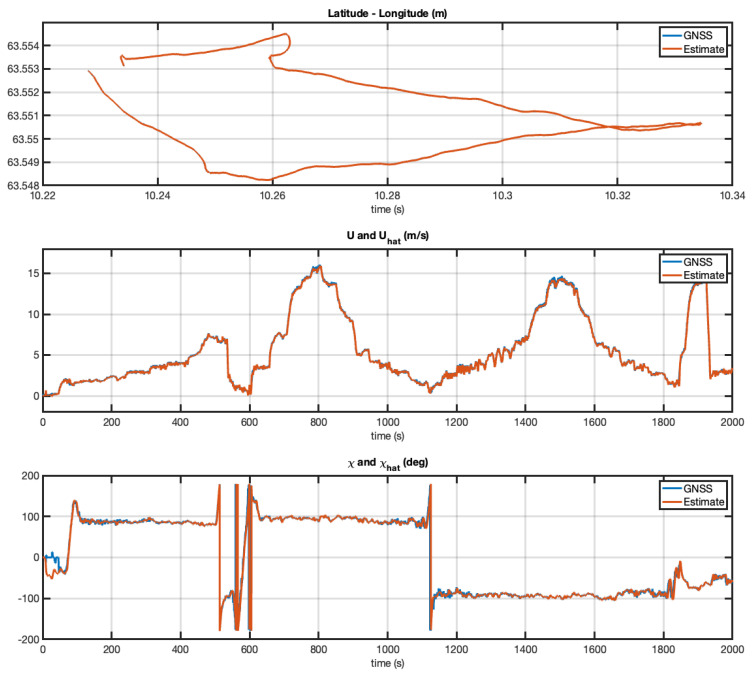
Experiment with the Mariner USV. Estimated SOG and COG versus time using latitude and longitude measurements at 5 Hz. Both the GNSS receiver and the EKF estimate the SOG and COG quite well for speeds higher than 1 m/s. The only discrepancy is during start up (low speed).

It should be noted that the GNSS values for SOG and COG were not validated. Hence, they do not represent groundtruth. Because of this, Figure 8 only shows the difference between the two algorithms. The GNSS receiver determines the distance between two fixes, and by using the time taken to travel this distance it can deduce its speed. The COG and SOG can therefore seem erratic under certain conditions. For example, when the USV was moving slowly through rough seas, the antenna moved from side to side as well as in the direction of the vehicle. In contrast, the EKF estimates were not affected by this.

The Kalman filter covariance matrices were chosen as $Q_{d}=\mathrm{diag}\left\{1\times 10^{7},1\times 10^{3}\right\}$ and $R_{d}=1\times 10^{-8}\;\mathrm{diag}\left\{1,1\right\}$. Figure 8 shows the performance of the five-state EKF. The EKF succeeded in estimating both the SOG and COG with good accuracy.

## 7. Experiments with the Otter USV

**Figure 9:**
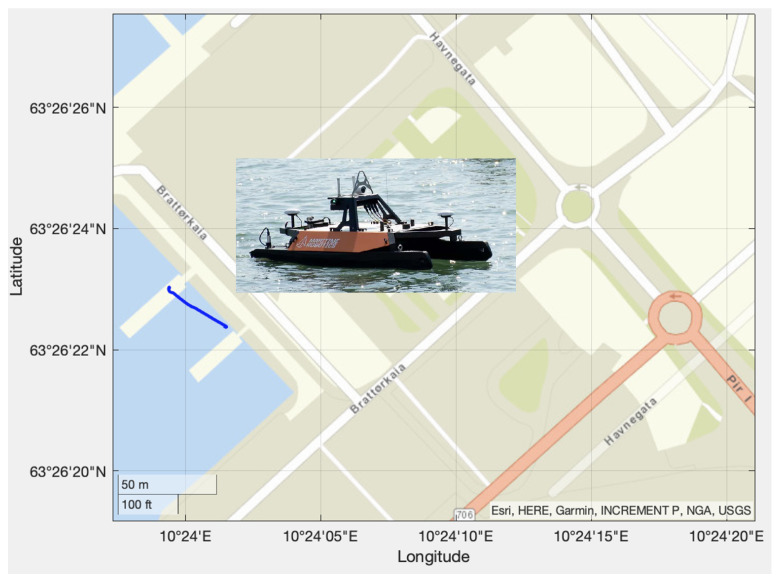

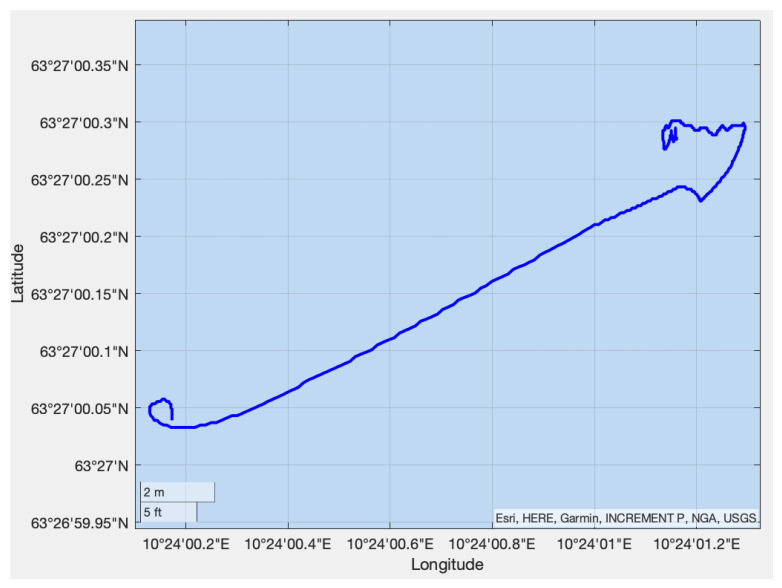
The blue line shows the path of the Otter USV when operating in the harbor (upper plot) and in the middle of the Trondheim fjord (lower plot). The starting points are $63^{\circ }\;26^{\prime }\;22.37^{\prime \prime }\;N$ and $10^{\circ }\;24^{\prime }\;1.49^{\prime \prime }\;E$ and $63^{\circ }\;27^{\prime }\;0.04^{\prime \prime }\;N$ and $10^{\circ }\;24^{\prime }\;0.17^{\prime \prime }\;E$, respectively.

In the second experiment, the Otter USV was used. The USV’s location is shown in Figure 9. The blue line indicates the traveled path, which is in the proximity of the Maritime Robotics main office in the Trondheim harbor, Norway. The experiments were performed in sea state 1 corresponding to wave amplitudes below 0.1 m. The Otter USV is a much smaller vehicle than the Mariner USV and it typically operates at very low speed (0–2 m/s). This is challenging for the state estimator since the course angle is not defined at zero speed. However, the EKF was stable even at zero speed thanks to the non-zero Singer constants $\alpha_{1}$ and $\alpha_{2}$ in the model. Latitude and longitude were measured using a u-blox NEO-M8Q GNSS receiver at 5 Hz [15], while the SOG and COG measurements were complementary measurements used to benchmark the EKF. The filter covariance matrices were chosen as $Q_{d}=5\times 10^{5}\;\mathrm{diag}\left\{1,1\right\}$ and $R_{d}=1\times 10^{-8}\;\mathrm{diag}\left\{1,1\right\}$.

**Figure 10:**
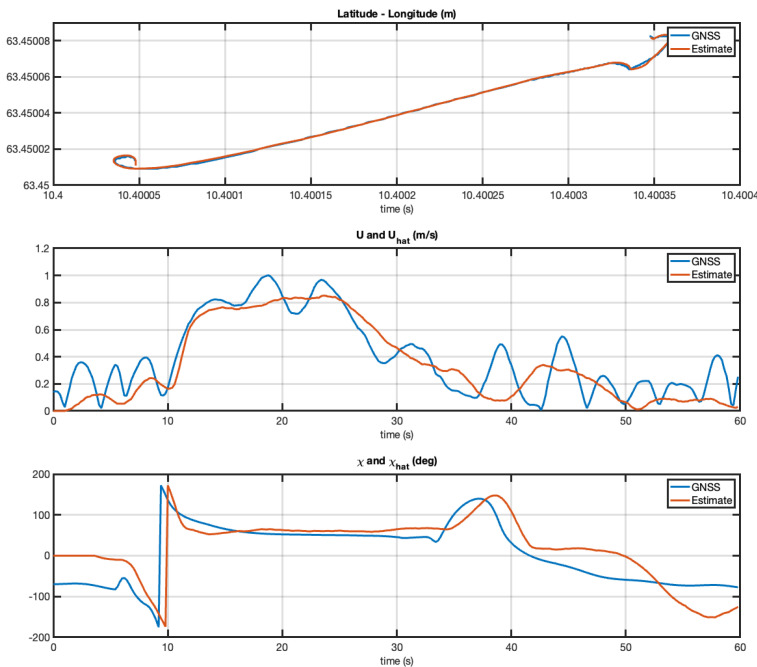
Experiment with the Otter USV in the middle of the Trondheim fjord. Estimated SOG and COG versus time using latitude and longitude measurements at 5 Hz.

**Figure 11:**
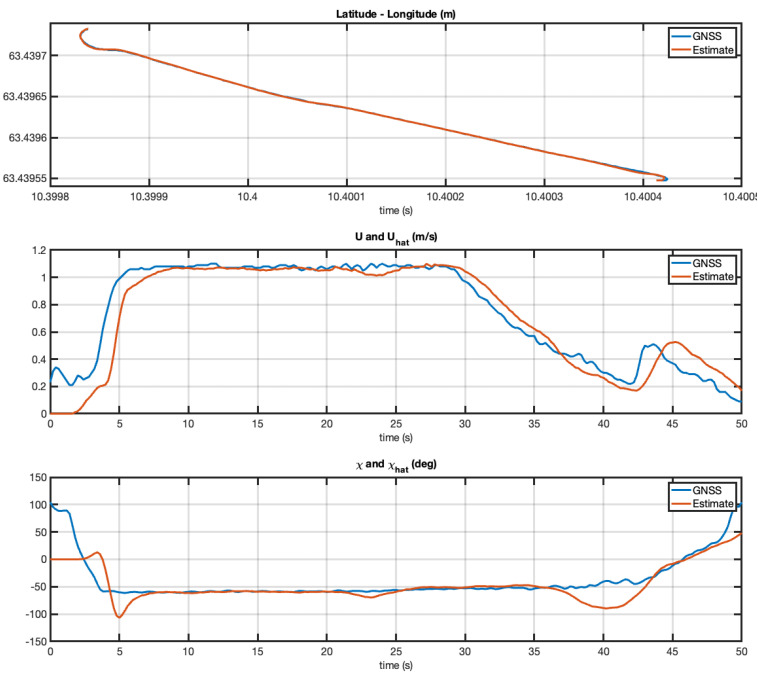
Experiment with the Otter USV in the harbor. Estimated SOG and COG versus time using latitude and longitude measurements at 5 Hz. The results are good when the vehicle moves at approximately 1.0 m/s and less accurate at very low speeds.

The experiment with the Otter USV was repeated, but, the second time, the vehicle was taken out of the harbor to operate in the middle of the Trondheim fjord. However, similar performance was achieved as seen from the plots in Figure 10 and Figure 11. From this it can be concluded that the EKF works very well at forward speed while it reaches an arbitrary course angle at zero speed. This is expected since the course angle is not defined at zero speed.

## 8. Discussion

The experiments with the Otter and Mariner USV systems confirm that the COG, SOG, and course rate can be estimated from latitude and longitude measurements with great accuracy when the speed is above a certain threshold value (typically 0.5 m/s). The experiments also confirm that the speed estimates are less accurate close to zero speed. At zero speed the course angle is not defined. Hence, the course angle estimate will converge to an arbitrary angle in the interval [−π,π). However, the EKF is exponentially stable at zero speed thanks to the Singer constants in the filter.

## 9. Conclusions

The main result of the article is a five-state extended Kalman filter (EKF) aided by GNSS latitude-longitude measurements for efficient estimation of course over ground (COG), speed over ground (SOG), and course rate. This is of particular interest for unmanned surface vehicle (USV) systems equipped with low-cost navigation sensor suites. For such systems, a gyrocompass is too expensive compared to the cost of the vehicle. A magnetic compass is unreliable due to electromagnetic interference caused by propellers and thrusters. A dual-antenna GNSS system on the same receiver (RTK GNSS) is an alternative, but the operational reliability (number of dropouts) depends on the density of the reference-station network, number of available satellites, multipath, ionospheric disturbances, etc. Furthermore, it has been demonstrated that the five-state EKF can estimate the SOG, COG, and course rate of a USV quite accurately using a single low-cost u-blox GNSS receiver. It has also been shown that the state estimates can be used to implement course and path-following control systems onboard the USV. The performance of the five-state EKF has been experimentally verified by using navigational data from two commercial USV systems, the Otter and the Mariner USVs by Maritime Robotics, which were operated west of Trondheim, Norway. The results from the experiments demonstrated that the EKF could estimate all states from low-cost latitude–longitude measurements.

## Figures and Tables

**Table 1:** Discrete-time EKF for SOG, COG, and course rate estimation.

Initial values	$\;{\hat{x}}^{-}\left[0\right]=x_{0}$
	${\hat{P}}^{-}\left[0\right]=E\left[\left(x\left[0\right]-{\hat{x}}^{-}\left[0\right]\right){\left(x\left[0\right]-{\hat{x}}^{-}\left[0\right]\right)}^{⊤}\right]=P_{0}$
Kalman filter gain matrix	$K\left[k\right]={\hat{P}}^{-}\left[k\right]C_{d}^{⊤}\left[k\right]{\left(C_{d}\left[k\right]{\hat{P}}^{-}\left[k\right]C_{d}^{⊤}\left[k\right]+R_{d}\left[k\right]\right)}^{-1}$
State vector corrector	$\hat{x}\left[k\right]={\hat{x}}^{-}\left[k\right]+K\left[k\right]\left(y\left[k\right]-h({\hat{x}}^{-}\left[k\right])\right)$
Covariance matrix corrector	$\hat{P}\left[k\right]=\left(I-K\left[k\right]C_{d}\left[k\right]\right){\hat{P}}^{-}\left[k\right]{\left(I-K\left[k\right]C_{d}\left[k\right]\right)}^{\;\;⊤}+K\left[k\right]R_{d}\left[k\right]K^{\;⊤}\;\left[k\right]$
State vector predictor	${\hat{x}}^{-}\left[k+1\right]=A_{d}\;\hat{x}\left[k\right]+B_{d}u\left[k\right]$
Covariance matrix predictor	${\hat{P}}^{-}\left[k+1\right]=A_{d}\hat{P}\left[k\right]A_{d}^{⊤}+E_{d}Q_{d}\left[k\right]E_{d}^{⊤}$

## Appendix A. Matlab Function: EKF5states.m

It is straightforward to implement and use the five-state EKF in a practical implementation. The Matlab [16] function EKF5states.m listed below can be used as template for coding in other languages. The function should be implemented in a loop or a real-time system according to:
